# Study protocol for coaching and leadership in autism support settings: a cluster randomized controlled hybrid type 2 effectiveness-implementation trial

**DOI:** 10.1186/s13012-026-01497-0

**Published:** 2026-03-28

**Authors:** Jill Locke, Nathaniel J. Williams, Aksheya Sridhar, Wendy Shih, Christine Espeland, Daina Tagavi, Karen Bearss

**Affiliations:** 1 University of California, Los Angeles, 457 Portola Plaza, Los Angeles, CA 90095 USA; 2 Boise State University, 1910 W. University Dr., Boise, ID 83725 USA; 3 University of Washington, Seattle, WA 98115 USA; 4 Catalight, Walnut Creek, CA USA

**Keywords:** Implementation leadership, Implementation climate, Implementation strategy, Educator coaching, Externalizing behavior, Autism, Schools

## Abstract

**Background:**

The increased prevalence of autism spectrum disorder creates a sense of urgency to improve outcomes for this population in publicly funded education systems, the primary setting in which autistic children receive behavioral health services in the United States. Important barriers to progress include a lack of feasible clinical interventions that address autistic children’s externalizing behaviors in schools and major challenges sustaining fidelity to newly implemented programs over time. This trial addresses these gaps by (1) testing the clinical effectiveness of the Research Units on Behavioral Interventions in Educational Settings (RUBIES) program relative to educator psychoeducation on externalizing behaviors of autistic children in public elementary schools, and (2) testing the effects of adding a leadership-focused organizational implementation strategy, Helping Educational Leaders Mobilize Evidence (HELM), to educator coaching in RUBIES on RUBIES sustainment.

**Methods:**

In a cluster-randomized, hybrid type 2 effectiveness-implementation trial, schools will be randomized to one of 3 arms: 1) educator coaching in RUBIES and school participation in HELM; 2) educator coaching in RUBIES only; or 3) a control condition incorporating an active clinical comparator, educator psychoeducation. We will enroll 42 schools and 126 educators yoked to 126 elementary-aged autistic children. Depending on arm, educators will complete study instruments up to six times: 1) Spring semester prior to the year of school and student enrollment (implementation baseline; arms 1–2); 2) Fall semester Year 1 (clinical baseline; arms 1–3); 3) 16 weeks (arms 1–3); 4) 24 weeks (arms 1–3); 5) 52 weeks (arms 1–2); and 6) 76 weeks (arms 1–2). The primary clinical outcome compares arms 1 & 2 vs. 3 on change in autistic children’s externalizing behavior from clinical baseline to 24 weeks. The primary implementation outcome compares arms 1 vs. 2 on RUBIES sustainment, operationalized as educators’ average RUBIES fidelity at 52 and 76 weeks.

**Discussion:**

Generating evidence for the clinical effectiveness of RUBIES addresses a significant gap in educator-delivered interventions to minimize highly prevalent externalizing behaviors among autistic children in public schools. Simultaneously, testing the effectiveness of HELM on sustainment of RUBIES will inform future efforts to successfully implement and sustain new innovations for autistic youth in public schools.

**Name of the registry:**

Clinical Trials.

**Trial registration:**

NCT07276750.

**Date of registration
**

12/10/25.

**URL of trial registry record**

https://clinicaltrials.gov/study/NCT07276750?cond=Autism&intr=RUBIES&rank=1.

Contributions to the literature

- Sustainment of evidence-based practices in schools is challenging given the implementation cliff that occurs oversummer break in-between school years. This study will simultaneously evaluate the clinicaleffectiveness of a promising, relatively low-cost, and highly feasible intervention that addressesexternalizing behavior in autistic students, called RUBI in Educational Settings (RUBIES), and theimplementation effectiveness of a promising leadership-focused implementation strategy calledHelping Educational Leaders Mobilize evidence (HELM), in public elementary schools.
- The proposed trial will generate evidence regarding the clinical effectiveness of RUBIES which fills agap in educator-delivered interventions that meaningfully support autistic youth throughout theschool day to minimize externalizing behaviors.
- Leadership-focused implementation strategies have shown promise for improving fidelity in othersettings; however, their effects on sustainment and in public schools remains unexamined. Theproposed trial tests whether the addition of HELM improves sustainment of evidence-based practicesin public elementary schools beyond educator coaching alone.
- If successful, this study will have substantial public health impact because it will produce an effectiveintervention for a prevalent problem among a high impact population and will determine how tosustain this (and other) intervention(s) with high fidelity.

## Background

Autism is a complex and lifelong developmental disorder that affects 1 in 31 children in the United States [1, 2]. The high and increasing prevalence of autism places a significant obligation on public education systems [3, 4]. For example, in the U.S., public schools are the primary setting in which autistic children receive behavioral health interventions, partly due to federal laws (e.g., Individuals with Disabilities Education Act) that require supports for children with disabilities [3, 4]. However, most educators receive limited training, resources, and support for working with autistic students, contributing to poorer academic, behavioral, and social outcomes for autistic children [5].

Autistic children commonly exhibit externalizing behavior (e.g. physical/verbal aggression, property destruction, transition difficulties) in schools, which significantly hampers their participation in less restrictive environments [6–10]. Externalizing behavior persists if unaddressed, and is a major contributor to educator burnout and turnover [11, 12]. Moreover, these challenges have additive short-term (e.g. increased rates of physical restraint, seclusion, suspension, expulsion) and long-term impacts (e.g. dropout, trauma, exacerbated mental health issues) [10, 13, 14]. Therefore, there is a critical need to increase the feasibility, efficiency, and effectiveness with which autistic children’s externalizing behaviors are addressed in classrooms.

There are currently no widely disseminated evidence-based interventions for educators to effectively and efficiently address externalizing behavior of autistic children in schools. The Research Units on Behavioral Interventions in Educational Settings (RUBIES) intervention is a promising intervention designed to help educators functionally evaluate and understand autistic children’s externalizing behavior as communication, and to account for autistic characteristics (e.g. rigidities and sensory sensitivities) in responding to that behavior [15]. RUBIES was adapted from RUBI, an empirically-validated caregiver-mediated behavioral intervention for autistic children with co-occurring externalizing behaviors [16, 17]. The adapted RUBIES intervention includes 9 modules and provides a simple framework that helps teach educators how to identify the function of a child’s behavior in order to apply a tailored positive behavioral support strategy that addresses the behavior’s function [18–20]. Previous studies indicate RUBIES is feasible, acceptable, and appropriate for schools; however, its clinical effectiveness in reducing child externalizing behaviors in schools has yet to be evaluated [21].

Clinical interventions such as RUBIES cannot meaningfully improve population health if they are not implemented with fidelity (i.e., the extent to which the intervention is implemented as designed) and sustained (i.e., the degree to which the intervention continues to be delivered with fidelity in a setting) over time [22, 23]. Malleable organization-level factors, including leadership, culture and climate, and operating policies and procedures, frequently stymie efforts to implement autism interventions with fidelity in schools and sustain them over time [24–26]. Previous research has highlighted two related malleable organizational factors that are particularly effective in predicting evidence-based practice (EBP) fidelity: implementation leadership (i.e., specific leader behaviors that support EBP use) and implementation climate (i.e., shared perceptions among intervention users of the extent to which EBP use with fidelity is expected, supported, and rewarded within the setting) [24, 27–29]. Research indicates that implementation strategies targeting implementation leadership and climate can improve fidelity to and the effectiveness of clinical interventions, as well as create an environment that is conducive to EBP sustainment over time [30–32].

Although organizationally focused implementation strategies have shown promise, schools represent a unique context, requiring adaptations to the form of organizational strategies as well as unique tests to confirm these strategies are effective within school settings. For example, the perpetual shifts in staff, students, operating policies and procedures, and emergent crises that plague public schools require specific modification to support high-fidelity EBP use to achieve positive outcomes for students [33]. To address this gap, we used the Discover, Design/Build, and Test framework [34] to collaboratively redesign, with school leaders and staff, one of the most promising organizational implementation strategies called Leadership and Organizational Change for Implementation (LOCI). The LOCI strategy targets general and implementation-specific leadership to improve implementation climate for a specific EBP [35]. LOCI includes a) data-based feedback; b) coaching of first-level leaders to enhance transformational and implementation leadership, and c) strategy meetings with executive leaders to improve implementation climate [36, 37]. Studies show that LOCI improves implementation leadership and climate, as well as EBP fidelity in mental health and substance abuse settings; however, this strategy has not previously been systematically adapted to improve EBP sustainment in school settings [35, 38].

Our collaboratively redesigned, school-focused organizational implementation strategy was called HELM for Helping Educational Leaders Mobilize evidence. HELM works with school building leadership teams (including the school principal and assistant principal) to improve the team’s implementation leadership and ability to develop a supportive implementation climate, with the ultimate goal of supporting EBP fidelity and sustainment within the school context. Results from a pilot study evaluating HELM indicated it was feasible and acceptable to school principals and staff and significantly improved implementation leadership and climate compared to an implementation attention control condition [39]. However, the effects of HELM on EBP fidelity and sustainment have not yet been tested.

## Study purpose and aims

The purpose of this study is to test both RUBIES and HELM in a large-scale cluster-randomized, hybrid type 2 effectiveness-implementation trial and to evaluate the mechanisms through which these interventions influence their targeted outcomes [40]. In the trial, schools will be randomized to: Arm 1) educator coaching in RUBIES and school leadership participation in HELM; Arm 2) educator coaching in RUBIES only; or Arm 3) an active clinical comparator, educator psychoeducation. The primary clinical comparison contrasts arms 1 and 2 versus arm 3 on improvement in autistic youths’ externalizing behavior. The primary implementation comparison contrasts arms 1 versus 2 on RUBIES sustainment. If successful, this study will identify an effective clinical intervention for reducing externalizing behavior of autistic children in schools (RUBIES) and an organizational implementation strategy specifically designed to sustain RUBIES and other EBPs in schools with high fidelity (HELM). Because HELM can be applied to a range of EBPs, results are expected to generalize with strong potential for scale up and public health impact.

**Fig. 1:**
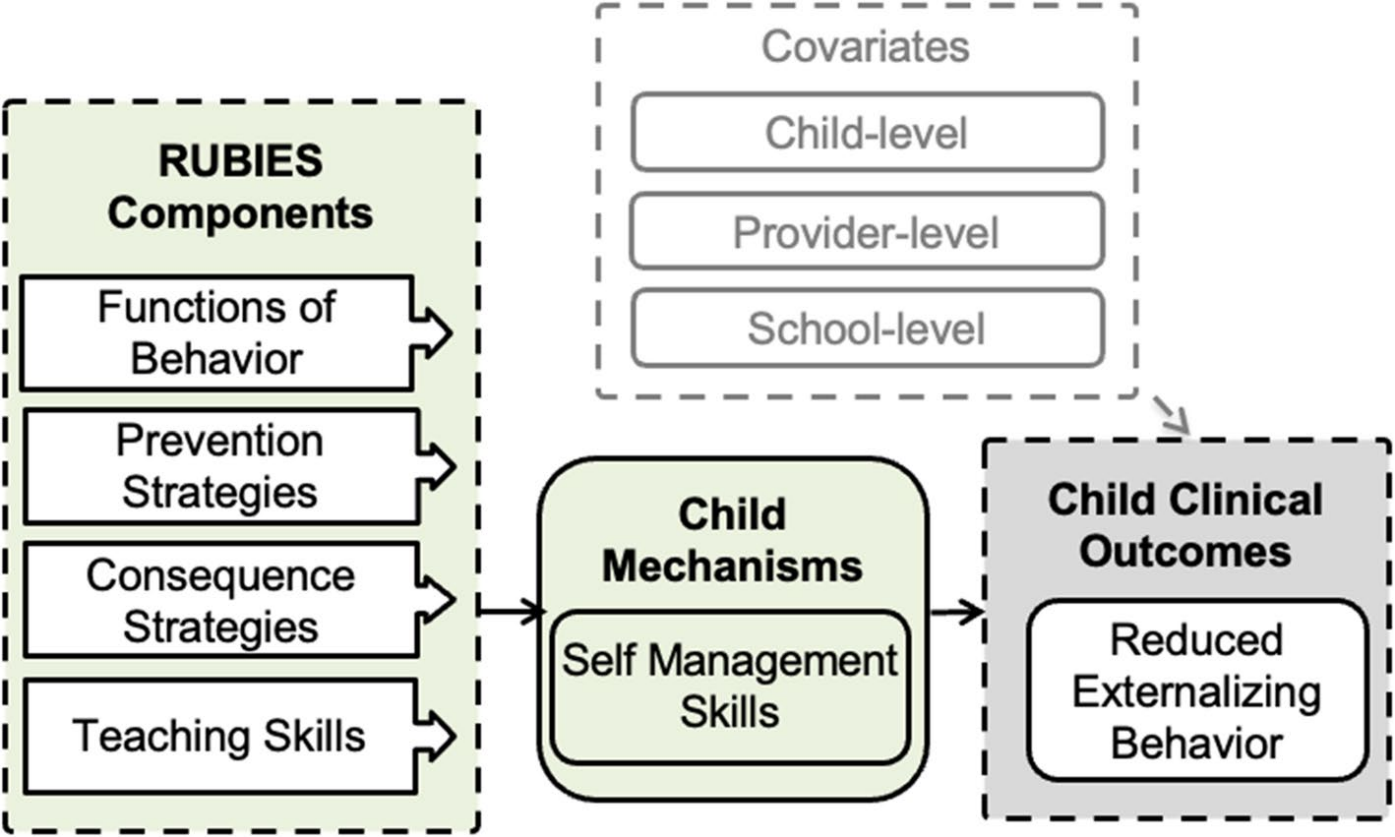
RUBIES components, hypothesized mechanisms, and target outcomes

**Fig. 2:**
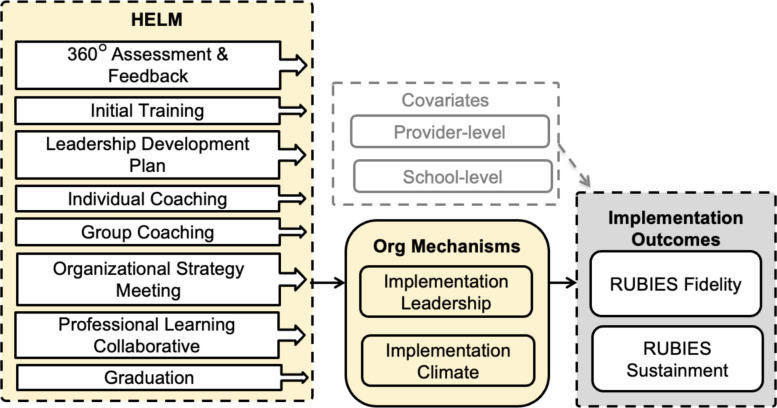
HELM components, hypothesized mechanisms, and target outcomes

Figures 1 and 2 depicts core HELM and RUBIES components, their respective mechanisms of change, and targeted outcomes. Evaluation of implementation mechanisms is critical to developing effective and streamlined implementation strategies [41, 42]. In our conceptual model, we theorize that RUBIES will activate the *child-level mechanism* of self-management skills which will impact the clinical outcome of externalizing behavior. We also theorize that HELM will activate *organizational mechanisms* of implementation leadership and climate which will impact the implementation outcome of sustainment.

The trial tests the following hypotheses:

### Hypothesis 1

During Year 1 of school enrollment, RUBIES will significantly reduce autistic children’s externalizing behavior from clinical baseline to 24 weeks compared to educator psychoeducation (arms 1 & 2 vs. arm 3).

### Hypothesis 2

During Year 2 of school enrollment, educator sustainment of RUBIES (operationalized as level of educator fidelity to RUBIES through the second year of study enrollment) will be superior in schools that receive HELM relative to schools that do not receive HELM, 52 and 76 weeks after clinical baseline (arm 1 vs. arm 2).

### Hypothesis 3a

Increases in autistic children’s self-management skills will mediate RUBIES’ effect on autistic children’s externalizing behavior relative to educator psychoeducation (arms 1 & 2 vs. arm 3).

### Hypothesis 3b

Increases in principal implementation leadership and school implementation climate will mediate HELM’s effect on educator sustainment relative to RUBIES coaching alone (arm 1 vs. arm 2).

## Method

### Study design

**Fig. 3:**
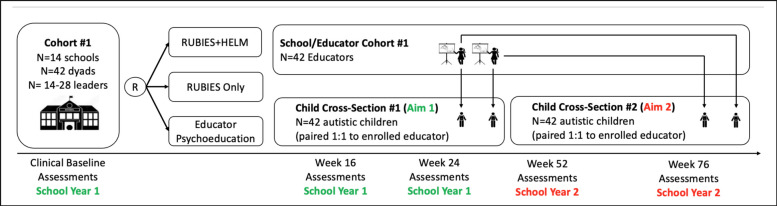
Study design illustrated with cohort 1

This study will use a hybrid type 2 effectiveness-implementation trial with HELM, RUBIES, and a psychoeducation only control condition to provide a rigorous test of the effects of RUBIES and HELM across 126 educators paired with 126 autistic children in 42 elementary schools. The full sample of 42 elementary schools will be enrolled in three cohorts of approximately 14 schools each nationwide. Each cohort will participate for two years. Depending on arm, educators will complete study instruments up to six times: 1) Spring semester prior to the year of school and student enrollment (implementation baseline; arms 1–2); 2) Fall semester Year 1 (clinical baseline; arms 1–3); 3) 16 weeks (arms 1–3); 4) 24 weeks (arms 1–3); 5) 52 weeks (arms 1–2); and 6) 76 weeks (arms 1–2). Leaders and educators will be compensated $40 at implementation and clinical baseline, $50 at Week 16, $60 at Week 24, $70 at Week 52, and $80 at Week 76 for the completion of study instruments at each timepoint. See Fig. 3 for study design.

### Randomization

The biostatistician (WS) will generate the random allocation sequence; all other research team members will not have access to the randomization sequence. Schools in each cohort will be randomized (1:1:1) to one of three parallel arms (*n* = 14 schools per arm): 1) RUBIES + HELM; 2) RUBIES only; 3) or educator psychoeducation. Randomization will be stratified based on median split for: 1) size of school at the time of enrollment; and 2) number of possible dyads (identified at screening for each enrollment cohort). Study enrollment, randomization, and engagement for all schools begins in the Spring prior to Year 1. Schools randomized to psychoeducation will be enrolled in the trial for one year; schools randomized to either RUBIES + HELM or RUBIES only will be enrolled in the trial for two years; children will be enrolled for one year.

### Participants

Inclusion criteria for schools are: 1) no previous RUBIES or HELM exposure; 2) identification of at least two autistic children with co-occurring externalizing behavior prior to study enrollment, 3) principal/administrator agreement to randomization, and 4) principal/administrator agreement to participate in HELM, if assigned to that condition.

### Leaders and educators

An anticipated total of 373 participants from 42 public elementary schools in the USA will be enrolled in the proposed study. This includes 126 educators who will receive either RUBIES or educator psychoeducation and 126 autistic children yoked to those educators. It also includes 28 first-level leaders, 9 district administrators, and 84 additional educators (3 per school) who will complete 360° assessments of school leadership and climate in the HELM and RUBIES only conditions over the course of the study. Participants will remain in the study for two years, unless they are randomized to psychoeducation (enrollment concludes at the end of Year 1) or withdraw or change jobs.

Educators are eligible for inclusion if they are special or general education teachers, paraeducators, or other staff (e.g., speech therapist, school psychologist) at a public elementary school who provide direct instruction and/or behavioral support to autistic children during the school day. For the 28 schools randomized to RUBIES + HELM or RUBIES only, approximately three additional educators (e.g., teachers, paraeducators, specialists, counselors) involved with autistic students will be recruited per school to complete the 360° assessments of implementation leadership and climate at each time point (*n* = 84 additional educators), one to two principals or school administrators will be recruited per school, and one to two district administrators will be recruited per district. District leaders will be requested not to use HELM strategies with participating schools randomized to other conditions. No participants will be excluded based on sex, age, or racial/ethnic background.

### Autistic children

Enrolled educators will identify one autistic child with whom they currently work for potential enrollment in the trial. Educators will share information about the study with the child’s caregiver/legal guardian and ask to share the caregiver’s contact information with the research team to complete informed consent. Consenting caregivers will be contacted by the research team. All study activities will be described verbally and in writing during the caregiver informed consent process. The research staff will describe the study and expected roles and responsibilities, risks and benefits, and confidentiality procedures and will obtain electronic parent/guardian consent. Autistic children enrolled in the trial will be paired with one educator (1:1 dyad). We anticipate recruiting at least two autistic children per school (range 2–5 children per school). Inclusion criteria for children are: 1) a documented autism diagnosis via school records (i.e., Individualized Education Program; IEP); 2) enrolled with a participating educator; 3) in grades K-5; ages 5–12; and 4) a Sutter Eyberg Student Behavior Inventory-Revised (SESBI-R) [43] total score ≥ 101 and/or an Emotion Dysregulation Inventory [44] sum score of ≥ 8 at baseline as rated by the educator after the dyad consents. Educators will be excluded if their principal/administrator declines participation. If an educator serves no autistic children who meet eligibility criteria, the educator will be excluded.

### Procedures

#### Recruitment

Recruitment of public elementary schools will occur in multiple stages – the research team will: (1) meet with school district officials to obtain a list of elementary schools that serve autistic children in Kindergarten through fifth grade; (2) contact the principal at each prospective school to share information about the study and set up an initial meeting; and (3) work with school district officials and principals to set convenient times to deliver RUBIES and HELM, if randomized to those conditions. All participants will provide electronic informed consent. Schools will be compensated $400 to support enrollment and allow educators time to complete study activities.

## Clinical interventions

### Rubies

RUBIES is a manualized, 9-session educator-delivered intervention that addresses externalizing behaviors in autistic children [45, 46]. Although RUBIES follows a standardized manual, behavioral strategies are tailored to the autistic student, informed by target behaviors and their hypothesized function, as well as child- and educator-specific classroom needs. One goal of RUBIES is for educators to build mastery over a set of behavioral skills that are used to support the student currently under their care with potential to generalize to other autistic students in their classrooms. RUBIES also focuses on engaging the student’s broader educational team through an initial collaborative planning session as well as targeted goals in every session focused on activating a weekly team communication and generalization plan. Sixteen weeks will be allotted to complete the 9 modules, to account for cancellations, scheduling conflicts, holidays, etc. Educators will receive a RUBIES workbook, access to an online platform that houses resources (e.g., extra copies of activity sheets, video models of an educator implementing RUBIES strategies), and a toolkit with recommended sensory tools and premade visual supports to accompany RUBIES use. Educators will engage in RUBIES sessions with a coach who has a master’s degree or higher and completed training to fidelity in RUBIES (i.e., manual review, viewing of RUBIES sessions, co-leading sessions with a certified RUBIES-trainer). The coach will facilitate weekly hour-long RUBIES sessions with educators via Zoom at a convenient time. Coaching sessions will include a practice plan review, didactic instruction on behavioral strategies, and coaching and consultation to tailor newly learned strategies. Educators will be offered a Booster session at three time points during Year 02. Booster sessions will involve reviewing three main components of RUBIES: conducting a functional assessment, prevention strategies, and how to implement more effective responses to challenging behaviors.

### Educator psychoeducation

Autistic children in schools assigned to the educator psychoeducation condition will receive standard of care school-based interventions (no research intervention). Participating educators in schools randomized to this condition will receive an online, self-paced webinar program via a web-based learning portal. The program includes 8-modules on supporting autistic children in schools, without discussion of behavioral management strategies: 1) Introduction to Autism, 2) Autism in Schools, 3) Interventions for Supporting Communication, 4) Executive Functioning, 5) Inclusion in Schools, 6) Autism EBPs, 7) Social Functioning in Autism, and 8) Recess Engagement Strategies. Each module is 20–35-min long.

### Implementation strategies

#### HELM

Members of the research team will work with school principals and district-level leaders who participate in HELM over the course of 9 months [33]. HELM includes eight components: 1) Assessment and Feedback. Principals, assistant principals, and educators familiar with RUBIES will complete an initial 360º survey on RUBIES implementation leadership and climate in the spring semester prior to the year of enrollment (HELM baseline, Year 0), Weeks 16, 24, 52, and 76. A detailed feedback report will be shared with the leader, to create a personal leadership development plan to support implementation and sustainment; 2) Initial Training. Principals, assistant principals, and district leaders will participate in didactic and interactive trainings on developing strategic implementation leadership behavior and an EBP implementation climate in their schools in the summer prior to Year 01 of study enrollment; 3) Leadership Development Plan. Principals will work with their coach to review their 360º assessment data and develop goals for improving implementation leadership and climate during the initial training and during coaching; 4) Individual Coaching. A former principal will provide monthly 1-h one-on-one coaching sessions via Zoom with the principals and assistant principals to review progress and update the leadership development plan. 5) Group Coaching. A former principal will offer optional monthly 1-h group coaching calls with HELM principals to review progress and share strategies across schools; 6) Organizational Strategy Meeting. Two, 1-h meetings with principals and district-level leaders will be held in each semester of Year 01, to develop and update an organizational implementation strategy plan. This meeting will provide a structured discussion of alignment between school- and district-level efforts to support RUBIES implementation and sustainment; 7) Professional Learning Collaboratives. Two professional learning collaboratives will be held with principals in Year 01 to review content (align HELM strategies with principles from the National Educational Leadership Standards, and EBP sustainment) and share strategies; and 8) Graduation. Principals’ final feedback will be reviewed, progress for the past year will be celebrated, and sustainment plans will be reviewed.

### Measures

Data will include quantitative surveys, qualitative interviews, and independent evaluator assessments over two years for each cohort (see Table 1).

**Table 1:** Study measures

			**Spring YR 0**	**Year 1**			**Year 2**	
	**Focus**	**Data source**	**Implementation** **screen/baseline**	**RUBIES screen/baseline**	**Week 16**	**Week** **24**	**Week 52**	**Week** **76**
**School**								
Characteristics	Characterization	Record review	X					
RUBIES School Implementation Leadership Scale*	HELM Mediator	HELM Educator	X		X	X	X	X
RUBIES School Implementation Climate Scale*	HELM Mediator	HELM Educator	X		X	X	X	X
**Student**								
Autism Classification	Characterization	Record review		X				
Demographics	Characterization	Parent		X				
School Services	Characterization	Educator		X	X	X		
SRS-2	Characterization	Educator		X				
SESBI-R	Primary Effective	Educator		X	X	X		
EDI	Secondary Effective	Educator		X	X	X		
CGI-Improve (Child Externalizing Behavior)	Secondary Effective	Educator + IE			X	X		
ABC-I	Secondary Effective	Educator		X	X	X		
CGI-Student Self-Management	RUBIES Mediator	IE			X	X		
**Educator**								
Demographics	Characterization	Educator	X					
RUBIES Educator BSP Implementation*	Primary Implement	Educator + IE			X	X	X	X
CGI-Sustain (Educator Sustainment of RUBIES Strategies)*	Secondary Implement	Educator + IE			X	X	X	X

^*^RUBIES and RUBIES + HELM Condition only

#### Demographics and context

School personnel will complete a demographic form in the Spring (Year 0). At clinical baseline of Year 1, Week 16, and Week 24, educators will document percent time and activities where the student is included in general education settings. School characteristics (e.g., school size, percent eligible for free lunch, racial/ethnic composition) will be obtained via school records in Spring of Year 0. Caregivers will complete a demographic form on students at baseline of Year 1.

 Autism Classification will be based on a documented autism diagnosis via school records (i.e., Individualized Education Program) collected at baseline of Year 1. Additionally, at baseline Year 1, educators will complete the Social Responsiveness Scale-2 (SRS-2,) a 65-item rating scale that measures social behavior associated with autism [47]. The SRS-2 has been used to distinguish autistic children from those with other psychiatric conditions.^107^The SRS-2 is used for children between the ages of 4–18 years with internal consistency of 0.88, inter-rater reliability of 0.75, and test–retest reliability of 0.88 [47].

#### Clinical outcomes

Student externalizing behavior will be rated at clinical baseline, Weeks 16, and 24. The Sutter Eyberg Student Behavior Inventory-Revised (SESBI-R; primary clinical effectiveness outcome) [43] is an educator-reported measure used to assess conduct problems in youth ages 2–16. It contains 38 items on which teachers indicate the current frequency of behavior problems (Never to Always) and indicate whether or not they find the behaviors problematic. The Intensity and Problem scales of the SESBI-R have demonstrated internal consistency coefficients of 0.98 and 0.96 and test–retest correlations of 0.87 and 0.93, respectively [48].

The Emotion Dysregulation Inventory [44] (EDI, secondary clinical effectiveness outcome) is a questionnaire that assesses emotion regulation and is validated for autistic youth. Educators rate the youth’s observed functioning over the past 7 days using a 5-point Likert scale from “not at all” to “very severe.” The EDI has excellent internal consistency (Cronbach’s alpha > 0.90) [49].

The Aberrant Behavior Checklist [50, 51] (ABC)-Irritability Subscale (secondary clinical effectiveness outcome) measures behavioral challenges in individuals with developmental disabilities. Educators rate items on a scale of 0 “never a problem” to 3 “severe problem.” Higher scores indicate greater problems. This study will utilize the Irritability subscale as it has been used in previous RUBI trials and is sensitive to change with intervention [16, 21]. The irritability subscale has excellent internal consistency (Cronbach’s alpha > 0.90) [52].

The Clinical Global Impressions-Improvement Scale [53] (CGI-I, secondary clinical effectiveness outcome), which is used widely in pharmacological and clinical trials to standardize assessment of overall change or improvement in targeted behaviors or outcomes [53, 54], is an assessment tool used to establish the improvement from baseline in defined intervention targets (e.g., focal behaviors) following treatment. The CGI-I is rated on a 7-point scale from 1 “very much improved”, to 4 “No Change” to 7 “very much worse.” Only scores of 1 and 2 will be demarked as a positive response to treatment. The CGI-I will be completed by a masked independent evaluator (IE) to assess improvement in student externalizing behaviors. CGI-I ratings are based on individualized narratives (intensity, frequency, duration) of student externalizing behavior provided by the educator via interview with the IE. The educator and IE then review and update the narrative at subsequent assessment points (Weeks 16 and 24). Using the updated narrative, along with other available information (educator ratings of behaviors on the EDI, SESBI-R and ABC-I), the IE completes the CGI-I rating. The CGI-I has been used in prior trials of RUBI and has been shown to be sensitive to detecting change in intervention-specific targets as well as differences between interventions [16, 17].

#### Implementation outcomes

Sustainment (primary implementation outcome) is defined as the level of educator fidelity to RUBIES through the second year of study enrollment (Weeks 52 and 76). Fidelity to RUBIES is operationalized on the RUBIES Educator Behavior Support Plan Implementation (BSPI) measure which will be rated by a trained independent evaluator masked to study condition. The BSPI is designed to assesses the educator’s current implementation of strategies that are detailed on the Behavior Support Plan, which is an organizing document that captures all RUBIES strategies developed for the educator’s student in collaboration with the RUBIES coach and implemented by the educator in Year 1. The IE rating on the BSPI will be completed at Weeks 16, 24, 52, and 76 and will be based on discussion with the educator around their current implementation of RUBIES strategies documented on their behavior support plan. Ratings on the BSPI are based on a scale of 1–8 (1 “none of the components of the behavior support plan are being implemented”; 4 “behavior support plan is partially implemented and with some implementation errors”; 8 “the behavior support plan is implemented consistently as intended in its entirety”). In a prior study of RUBIES, BSPI scores of educators randomized to RUBIES averaged 6.78 (SD = 1.25; scale of 1–8) [21].

The IE also will rate the Clinical Global Impressions-Sustainment scale (CGI-S, secondary implementation outcome), an adapted version of the Clinical Global Impressions-Improvement scale. The CGI-S will be rated by the IE on a 7-point scale from 1 “very much improved”, to 4 “no change” to 7 “very much worse.” To inform CGI-S ratings, a standardized questionnaire followed by IE interview will be conducted with educators at implementation baseline to establish a benchmark of the educator’s baseline skills. The questionnaire allows the educator to endorse whether, at baseline, they use any of 26 specific RUBIES strategies (yes/no response). The interview between IE and educator then focuses on characterizing the quality and frequency of endorsed strategies. Finally, the IE will use all information to characterize the educator’s overall skill mastery at baseline on a 7-point scale (1 “exceptional mastery”; 4 “developing mastery”; 7 “no demonstrated mastery”), which will allow the IE to standardize subsequent CGI-S ratings across educators who have variable foundations in behavioral strategies at baseline. At Weeks 16, 24, 52, and 76, the educator will update their current strategy “use” ratings and then meet with the IE to update characterization (quality/frequency) of the utilized RUBIES strategies. Using this updated information, the IE will complete the CGI-Sustainment rating (i.e., is the current use of educator RUBIES strategies 1 “very much improved”, 2 “much improved”, 3 “minimally improved”, 4 “no change”, etc. from baseline). RUBIES sustainment at Weeks 52 and 76 will be calculated as a binary variable: strategies “sustained” over time (Score of 1 or 2 – Much improved to very much improved from baseline) vs strategies not “sustained” over time (Score of ≥ 3: No Change to Very Much Worse from baseline).

#### RUBIES mediator

We hypothesize that RUBIES will result in reduced student externalizing behavior by increasing students’ use of self-management skills that are taught by their educator (e.g., use of a functional communication tool to request help/break, independent reference to visual support, engagement with self-regulatory strategy such as fidget, movement to calming corner). For example, functional communication training involves a teacher instructing the child on how to use a more functional communication request (e.g., “I need a break”). Successful reduction of challenging behavior is thus reliant, in part, on the child’s use of self-management skills. To assess this hypothesized mediator, student self-management skills will be rated at Weeks 16 and 24 by a trained independent evaluator (IE) masked to study randomization utilizing the Clinical Global Impressions-Self Management (CGI-SM) scale, an adapted version of the Clinical Global Impressions-Improvement scale [53, 54]. The CGI-SM will be rated by the IE on a 7-point scale from 1 (very much improved), to 4 (No Change) to 7 (very much worse). To inform CGI-SM ratings, a standardized questionnaire will be completed by the educator, followed by IE interview will be conducted at baseline. The questionnaire will allow the educator to endorse whether, at baseline, their student uses any of 16 specific self-management/self-regulation skills that are emphasized in RUBIES (yes/no response). The interview between IE and educator will then focus on characterizing the quality and frequency of the student’s use of endorsed strategies. At Weeks 16 and 24, the educator will update their student’s current strategy “use” ratings and then meet with the IE to update characterization (quality/frequency) of the utilized self-management strategies. Using this updated information, the IE will complete the CGI-SM rating (i.e., is the student’s current use of RUBIES self-management strategies are very much improved (1), much improved (2), minimally improved (3), no change (4), etc. from baseline). Effectively using self-management strategies will be calculated as a binary variable based on the CGI: effectively using self-management strategies (CGI Score of 1 or 2: Much Improved or Very Much Improved from baseline) versus not effectively using self-management strategies (CGI Scores ≥ 3: No Change to Very Much Worse from baseline).

#### HELM mediators

Educators will complete the School Implementation Leadership Scale (S-ILS) [55], a 21-item measure that assesses seven subscales of implementation leadership: knowledgeable (understanding of RUBIES and implementation issues), supportive (support for RUBIES use), proactive (anticipating and addressing challenges), perseverant (consistent and responsive to challenges), communicative (shares implementation related information with staff), has a vision/mission (oriented towards using RUBIES), and available in implementing RUBIES [5, 37]. The S-ILS is a psychometrically validated and reliable instrument (α = 0.95–0.98). Implementation leadership scores are generated for a school at each timepoint (implementation baseline, Weeks 16, 24, 52, and 76) by aggregating individual educator ratings to the school level.

Educators also will complete the School Implementation Climate Scale (S-ICS) [56], a 21-item measure that assesses seven subscales of implementation climate: focus, educational support, recognition, rewards, use of data, existing supports, and RUBIES integration [37, 56]. The S-ICS is a psychometrically validated and reliable instrument (α = 0.81–0.91; 39, 79). Implementation climate is scored by aggregating individual educator ratings to the school level.

#### Qualitative interviews

We will use a sequential (QUAN QUAL) mixed-methods design to understand discrepancies in implementation behavior (e.g., schools with high implementation leadership and low RUBIES fidelity and sustainment [57]. The functions are *sampling* (using quantitative data to identify the qualitative sample) and *expansion* (using qualitative data to provide depth and breadth of understanding of factors that contribute to outcomes that deviate from the theory of change), and the process is *connecting *(qualitative data will build on quantitative data) [57–59].

Interview questions will explore the implementation determinants and mechanisms [41, 60, 61]. Individual semi-structured interviews (approximately 30–45 min) will be conducted via Zoom at a convenient time for educators. The interview guide will be developed using the Exploration, Preparation, Implementation, and Sustainment framework [62] to examine multilevel determinants that explain what processes facilitated or hindered educators’ RUBIES sustainment. Interviews will be audio-recorded, then transcribed prior to coding. Interviews will be continuously conducted until we achieve thematic saturation.

### Data analysis

Preliminary data checks and analyses will screen for errors and explore normality, linearity, form, and outliers as well as psychometric characteristics of all latent constructs. Comparisons of intervention effects will use an intent-to-treat approach.

### Aim 1 analyses

Aim 1 evaluates the main effect of RUBIES vs. educator psychoeducation on child externalizing behavior among children in Year 1 of study enrollment. The primary outcome for this comparison is change in child externalizing behavior from baseline to Week 24, contrasting all groups assigned to RUBIES vs. educator psychoeducation (i.e., arms 1 & 2 vs. arm 3). We will fit a three-level Linear Mixed Model (LMM) as follows: the Level 1 model (piece wise time) with a “knot” (i.e., inflection point) at Week 16 used to capture each child’s change in externalizing behavior from Baseline to Week 16, and potentially different slope from Week 16 to Week 24. At Level 2 (child/educator; due to 1:1 pairing), each growth parameter will be modeled as a function of an intercept and child/educator-level covariates. We will select covariates by testing for differences across arms on youth and educator characteristics; covariates will be included if the effect size ≥|0.2|. At Level 3 (school), we will include a random intercept to account for the nesting of the child-educator dyads within schools and a treatment assignment indicator ([RUBIES + HELM] + [RUBIES only] vs. educator psychoeducation). We will test the interaction effect of treatment condition with time. Linear combinations of the growth parameters from this model will be used to test whether improvement in child externalizing behavior was superior for autistic children assigned to [RUBIES + HELM] + [RUBIES only] vs. educator psychoeducation from Baseline to Week 24.

### Aim 2 analyses

Aim 2 compares the effect of HELM vs. educator coaching on RUBIES sustainment as measured by BSPI scores (arm 1 vs. arm 2). The hypothesis states that intervening with HELM will induce better sustainment (i.e., higher RUBIES fidelity) in Year 2 at Week 52 and Week 76 compared to educator coaching only. The two observations of sustainment for each educator (paired 1:1 with a child) in Year 2 are nested within the educator, who is nested within a school. For this outcome, we will fit an LMM with the following hierarchical structure: the Level 1 model (observation) will include Week 52 and Week 76 fidelity observations for each educator. This level does not include a growth model because we do not assume fidelity will systematically increase or decrease during the sustainment school year; instead, we assume random fluctuation around an educator mean for the year. At Level 2 (educator), we will include a random intercept and educator-level covariates such as years of experience which may be a potential confounder. Covariates will be selected as described above. At Level 3 (school), we will include a random intercept and a treatment assignment indicator (RUBIES + HELM vs. RUBIES only) which will be contrast coded to facilitate main effect interpretations. The coefficient for the treatment assignment indicator in this model will test whether RUBIES sustainment (i.e., level of RUBIES fidelity in the second year of study enrollment) was superior for educators assigned to RUBIES + HELM compared to educators assigned to RUBIES only at Week 52 and Week 76. As a secondary implementation outcome, we will analyze a binary indicator of RUBIES sustainment at Week 52 and Week 76 (based on the CGI-S) to evaluate whether HELM improved the odds of sustaining RUBIES at a provisional ‘sustainment’ cutoff. This analysis will use a similar model as Aim 2, but data from different timepoints or distributions (LMM and Generalized LMM for binary outcomes).

Aim 3 involves mediation analysis to evaluate the mechanisms that link RUBIES to children’s externalizing behavior and HELM to RUBIES sustainment as measured by the BSPI. The primary focus will be testing the main effects of RUBIES on the hypothesized mediator, children’s self-management skills, and of HELM on its hypothesized mediators, implementation leadership and climate, in order to establish the discriminant effects of these exposures relative to their comparators. Following these primary analyses, exploratory analyses will be used to test the remainder of the mechanistic chain proposed by RUBIES. These analyses will use the product of coefficients approach to mediation and will build on the foundational Aim 1 model [63–66]. The same approach will be used for the HELM mediators, using the model for Aim 2 as the foundational model. Consistent with best practices, we will conduct robustness checks and sensitivity analyses regarding the sequential ignitability assumption and other assumptions of our mediation analyses following model estimation [67].

### Sample size and power

The primary focus of Aim 1 is to test the main effect of RUBIES (RUBIES + HELM and RUBIES only) vs. educator psychoeducation on change in children’s externalizing behavior from Baseline to Week 24. Assuming a Type-I error rate of 5% and SD of 6.2, a conservative within-person correlation in externalizing behavior of r = 0.3, 10% intraclass correlation (clustering within schools) for variance inflation and 20% attrition rate in children, a total of 126 autistic children paired 1:1 with educators (84 autistic children for RUBIES + HELM and RUBIES only vs. 42 children in educator psychoeducation) nested within 42 schools are needed to detect a difference of at least 3.8 points in the comparison of slopes in ABC-I with 80% power. A difference of 3.8 corresponds to a moderate standardized effect size of d = 0.62 in between groups change in children’s externalizing behavior and this effect size is clinically meaningful based on prior research [16]. The study assumed a modest clustering within schools of 10% because of the 1:1 pairing of the educator and autistic child. We expect 3 child-educator dyads per school to consent and enroll in the study. Hence, a total of 126/3 = 42 schools are needed.

For Aim 2, we will use only educators in the RUBIES + HELM vs. RUBIES only (84 educators within 28 schools) arms. With 84 educators as the effective sample size (42 each), nested within 28 schools, we have 80% power to detect a difference of at least 0.13 (or d = 0.76) in RUBIES sustainment (Week 52 and Week 76) between RUBIES + HELM vs. RUBIES only assuming SD = 0.17 based on pilot RUBIES data, a Type-I error rate of 0.05, 10% variance inflation for clustering within school effect, 25% attrition rate and within-person correlation of observations = 0.3. We assumed a higher attrition rate for educators since the turnover rate for educators are higher post pandemic.

For Aim 3, we focused the power calculation for the mediation aim on the effects of RUBIES on the hypothesized mediator, children’s self-management skills, in order to establish the discriminant effects of RUBIES relative to educator psychoeducation. Based on Heath et al., [68] children’s self-management skills have a moderately large effect for decreasing challenging behaviors (mediator on outcome). While we do not have preliminary data for the relationship between RUBIES on self-management skills, the power calculation is presented assuming small (0.25) and moderate (0.5) effects of RUBIES on self-management skills (treatment on mediator). With an effective sample size of 126 autistic children nested within 42 schools, we have 67% and 99% power to detect an indirect effect of RUBIES on children’s externalizing behavior assuming 1) small and moderate effects of RUBIES on self-management skills, respectively; 2) a moderate effect of self-management skills on decreasing externalizing behaviors; [68] 3) smaller effect of RUBIES on children’s externalizing behavior (Aim 1; treatment on outcome); and 4) Type I error rate of 0.05.

## Discussion

This study will evaluate the clinical effectiveness of RUBIES in schools and test a leadership-focused organizational implementation strategy to support *sustained*, high-fidelity delivery of an EBP for autistic children. This is the first fully powered trial of an evidence-based clinical intervention for externalizing behavior of autistic children (i.e., RUBI) that has been adapted for use by educators in elementary school settings. Externalizing behavior is a primary cause of autistic children’s exclusion from least restrictive environments, and educators often do not have the supports necessary to address these behaviors in inclusive settings [15]. As an educator delivered intervention, RUBIES will bypass long wait lists and reduce the need for treatment planning by specialized providers and allow externalizing behaviors to be more immediately addressed in the school setting. If RUBIES is clinically effective, it will address a significant need for educator-delivered interventions to minimize highly prevalent and debilitating externalizing problems among autistic children in schools. Validating this intervention will significantly advance the field and support its widespread use in schools to improve the health and well-being of autistic children.

Moreover, this is one of the few studies to measure sustainment of autism EBPs across school years. Sustainment is infrequently evaluated in implementation trials [69–71]. This study will examine implementation outcomes of cohorts of educators across two years to determine whether school participation in HELM results in greater RUBIES sustainment with fidelity over time and will use mixed methods to elaborate factors that contributed uniquely to sustainment with fidelity versus initial fidelity. Demonstrating the effectiveness of HELM on sustainment of RUBIES will address a highly prevalent and destructive barrier to improved population health: namely, abandonment or low-fidelity delivery of effective mental health interventions in schools.

Further, understanding the mechanisms of interventions and implementation strategies helps determine why an intervention or implementation strategy did or did not achieve its intended effects [41, 60]. Testing of RUBIES’ and HELM’s mechanisms will advance our understanding of theories underlying the etiology and treatment of externalizing behaviors in autism as well as mechanisms that support sustainment of effective health interventions in schools. Few EBP-agnostic implementation strategies that target ubiquitous organizational barriers have been developed. HELM was designed to be EBP agnostic and has the potential to be generalized to other EBPs in schools to improve implementation leadership and climate and ultimately support implementation and sustainment. Future research may further substantiate HELM with other interventions in schools and quantify the balance of efficiency and impact in a cost-effectiveness study. Because both interventions were developed and tested with local stakeholders, the resulting clinical intervention and implementation strategy are designed to be highly usable and scalable, which has significant implications for low-resource community contexts in which lay providers deliver mental health services. As of February 2026, no participants have been enrolled.

## Data Availability

The application described in this manuscript is freely available. Please contact the lead author for more information.

## Abbreviations

ABC: Aberrant Behavior Checklist
BSPI: Behavior Support Plan Implementation
CGI-I: Clinical Global Impressions-Improvement Scale
CGI-S: Clinical Global Impressions-Sustain
CGI-SM: Clinical Global Impressions-Self Management
EBP: Evidence-Based Practice
EDI: Emotion Dysregulation Inventory
HELM: Helping Educational Leaders Mobilize
IE: Independent Evaluator
LMM: Linear Mixed Model
LOCI: Leadership and Organizational Change for Implementation
QUAN: Quantitative
QUAL: Qualitative
RUBI: Research Units on Behavioral Interventions
RUBIES: Research Units on Behavioral Interventions in Educational Settings
SESBI-R: Sutter Eyberg Student Behavior Inventory-Revised
S-ICS: School Implementation Climate Scale
S-ILS: School Implementation Leadership Scale
SRS-2: Social Responsiveness Scale-2
